# Herbal and alternative medicine use: a cross sectional study to evaluate the prevalence and predictors of use in cancer patients

**DOI:** 10.3389/fphar.2025.1535795

**Published:** 2025-05-15

**Authors:** Mahmoud Al-Masri, Rula Darwish, Yasmin Safi, Aseel Mustafa, Dina Alzyoud, Mohammad Almasri, Bilal Kahhaleh, Mohammad Khader

**Affiliations:** ^1^ King Hussein Cancer Center, Department of Surgery, Amman, Jordan; ^2^ The University of Jordan, Faculty of Medicine, Amman, Jordan; ^3^ The University of Jordan, Faculty of Pharmacy, Amman, Jordan

**Keywords:** herbal medicine, alternative medicine, complementary therapies, cancer, prevalence, oncology care, public health

## Abstract

**Background:**

Herbal and alternative medicine is increasingly used among cancer patients to manage disease, symptoms, and improve quality of life. Reported usage rates worldwide vary from 3.5% to 90%. Understanding prevalence and predictors of such use is essential for safety and efficacy, especially considering potential interactions with conventional treatments.

**Methods:**

This cross-sectional study aimed to evaluate the prevalence and predictors of herbal medicine use among cancer patients in Jordan. All cancer patients, including pediatric and adult patients who can give consent or assent, were included in the study.

**Results:**

Out of 602 patients surveyed, 163 (27.1%) reported using herbal medicine. Among users, 68.7% were female, 52.9% had lower education levels, and 60% were unemployed. Of the key predictors: lower income (OR = 3.24, p = 0.021) and self-perceived knowledge of herbal medicine (OR = 18.9, p < 0.001). Knowledge assessment revealed that 54% relied on social media for information, while only 11% consulted healthcare professionals. Between 60% and 80% of patients were unaware of potential interactions between herbal treatments and cancer therapies. Additionally, the 85% reported that their healthcare providers did not inform them about these risks. Reasons for using herbs and alternative included maintaining health (52.4%) and cancer treatment (47.6%). Among non-users, 29% doubted its effectiveness, and 34% felt uninformed.

**Conclusion:**

The study revealed that low income and high self-perceived awareness about herbal medicine are key predictors of herbal usage. However, there is a significant knowledge gap, with many patients relying on social media and being unaware of potential interactions with oncology treatments. Customized educational interventions are needed to address these factors.

## 1 Introduction

The use of herbal and alternative medicine among cancer patients has gained increasing attention as a complementary approach to managing symptoms, enhancing quality of life, and even addressing the disease itself. While these practices can offer potential benefits, they also raise concerns regarding safety, efficacy, and possible interactions with conventional oncology treatments. This trend is part of a broader global shift towards holistic and patient-centered care, where individuals seek treatments that align with their cultural beliefs, personal preferences, and a desire for more natural or less invasive therapies. (Molassiotis et al., 2024; Boon et al., 2007; Viscuse et al., 2017; Sweiss et al., 2023).

The 2019 global report on traditional and complementary medicine by the World Health Organization (WHO) defines alternative or complementary medicine as a range of non-conventional treatment methods that are used as a replacement for standard medical treatments. (WHO global report on, 2019).

Studies have shown that the prevalence of herbal medicine use among cancer patients varies significantly across different populations and settings, with reported rates ranging from as low as 3.5% to as high as 90% (Oyunchimeg et al., 2017; Yeom and Lee, 2022). This wide range reflects differences in cultural practices, availability of herbal medicines, healthcare systems, and levels of awareness and education among patients. Despite the potential benefits, the use of herbal medicine alongside conventional cancer treatments raises significant concerns. These include the safety and efficacy of herbal medicines, the risk of adverse interactions with chemotherapy and other oncological treatments, and the lack of regulation and standardized quality control in the production of herbal supplements. (Kasprzycka et al., 2022; Cassileth and Deng, 2004; Posadzki et al., 2013).

In Jordan, as in many other countries, cultural beliefs and the accessibility of alternative treatments contribute to the popularity of herbal medicine. However, the reliance on non-professional sources for information, particularly social media, can lead to misinformation and increased risks, especially when herbal medicines are used concurrently with standard cancer therapies (Verhoeff and Steen, 2023). Despite these risks, the factors influencing herbal medicine use among cancer patients remain under-explored in this region, particularly in relation to socioeconomic status and perceived knowledge about these treatments. (Jalil et al., 2022; Abdel-Qader et al., 2020).

In 2010, F.U. Afifi et al. showed in their study that complementary and alternative medicine (CAM) use is common among patients with cancer in Jordan (Afifi et al., 2010).Another survey by Eran Ben-Arye et al. showed that herbal medicine use, which is prevalent in the Middle Eastern countries, has several potentially negative effects that include direct toxic effects, negative interactions with anticancer drugs, and increased chemo-sensitivity of cancer cells, requiring a reduction in dose density. Oncology Healthcare professionals (HCPs) working in countries in where herbal medicine use is prevalent need to better understand the implications of this practice (Ben-Arye et al., 2016).

There is a growing interest in usage of herbal and complementary medicine (HCM) alongside with conventional therapy worldwide and in Jordan. However, limited data exist on the prevalence and determinants of HCM use in Jordan among cancer patients (Ben-Arye et al., 2016). This study aims to address this gap by evaluating the prevalence, usage pattern, and key predictors of herbal medicine use. Herbal medicine includes herbs, herbal materials, herbal preparations and finished herbal medicines, containing as active ingredients parts of plants, or other plant materials, or combinations, whether used as a complementary therapy alongside standard cancer treatments or as an alternative treatment. Understanding these patterns will help healthcare providers identify target populations, tailor evidence-based recommendations, and develop patient education strategies to ensure the safe use of HCM in conjunction with conventional therapy.

## 2 Methods

### 2.1 Study design and population

This study employed a survey design to evaluate the prevalence and identify the predictors of herbal medicine use among cancer patients in Jordan. Sample size was calculated using Cochran’s formula for finite population, a final sample size of 602 patients was selected to enhance the study’s statistical power and ensure a comprehensive analysis of herbal medicine use and its predictors among cancer patients in Jordan. The study included a convenience sample of cancer patients receiving treatment at King Hussein Cancer Center in Jordan. Both adult and pediatric patients, who could provide informed consent or assent, were eligible for inclusion. The inclusion criteria were a confirmed cancer diagnosis, the ability to communicate in Arabic, and consent to participate in the study. Exclusion criteria included patients with cognitive impairments or those unwilling to participate.

### 2.2 Data collection

Data collection occurred in a baseline survey, a structured questionnaire was administered to 602 cancer patients to assess their use of herbal medicine. The questionnaire was developed based on previously validated tools in English language by Zahn et al. (2019) and included items on demographics (age, gender, education level, employment status, and income), clinical characteristics (cancer type, stage, and treatment modalities), and details about herbal medicine use (types of herbs used, reasons for use, sources of information, and perceived knowledge about herbal medicine). The questionnaire was translated into Arabic and circulated with experts before starting the data collection to assure the content and validity of the tool. To evaluate the validity of our scale further, a pilot study was conducted with a sample of 20 patients. The participants were administered the Herbal usage survey, and their responses were analyzed. The scale demonstrated good internal consistency, as indicated by a calculated Cronbach’s alpha value of 0.87. This suggested that the items within the scale are measuring a similar construct consistently. Overall, the combination of established scales, expert input, and the pilot study results provided initial evidence of the validity of our questionnaire.

### 2.3 Outcome measures

The primary outcome measures were the prevalence of herbal medicine use and the identification of predictors associated with its use. Predictors included demographic variables, clinical characteristics, self-perceived knowledge, and sources of information.

### 2.4 Statistical analysis

Descriptive statistics were used to summarize the baseline characteristics of the study population and the prevalence of herbal medicine use. Chi-square tests were employed to assess associations between categorical variables, while independent t-tests were used for continuous variables. Logistic regression analysis was conducted to identify predictors of herbal medicine use, with odds ratios (OR) and 95% confidence intervals (CI) calculated. A p-value of <0.05 was considered statistically significant.

### 2.5 Ethical considerations

The study was approved by the Institutional Review Board (IRB) of King Hussein Cancer Center (IRB#: 22 KHCC 006). All participants provided written informed consent or assent, as appropriate. The study adhered to the principles of the Declaration of Helsinki, ensuring the confidentiality and anonymity of all patient data.

## 3 Results

The study included a total of 602 cancer patients. The participants were predominantly female (62.0%) and married (66.9%). The majority had secondary education or less (49.3%) and were in the age group of 41–60 years (40.2%). The most common cancer diagnoses were breast cancer (30.2%) and hematologic cancers (15.9%), with the most participants were diagnosed with stage I (47.0%). Employment status varied, with 59.3% reporting no employment. (Table 1).

**TABLE 1:** Comparison between herbal users and non-users.

	Non-users (N = 439)	Users (N = 163)	Total (N = 602)	p value
Gender				0.038^1^
Female	261.0 (59.5%)	112.0 (68.7%)	373.0 (62.0%)	
Male	178.0 (40.5%)	51.0 (31.3%)	229.0 (38.0%)	
Social status				0.073^1^
Married	307.0 (69.9%)	96.0 (58.9%)	403.0 (66.9%)	
Single	91.0 (20.7%)	49.0 (30.1%)	140.0 (23.3%)	
divorced	12.0 (2.7%)	5.0 (3.1%)	17.0 (2.8%)	
widowed	29.0 (6.6%)	13.0 (8.0%)	42.0 (7.0%)	
Income levels				0.256^1^
1,000–500 JOD	140.0 (31.9%)	50.0 (30.7%)	190.0 (31.6%)	
Less than 500 JOD	248.0 (56.5%)	101.0 (62.0%)	349.0 (58.0%)	
More than 1000 JOD	51.0 (11.6%)	12.0 (7.4%)	63.0 (10.5%)	
Educational level				0.477^1^
Secondary or less	214.0 (48.7%)	83.0 (50.9%)	297.0 (49.3%)	
Higher education	170.0 (38.7%)	66.0 (40.5%)	236.0 (39.2%)	
Diploma	32.0 (7.3%)	10.0 (6.1%)	42.0 (7.0%)	
None	23.0 (5.2%)	4.0 (2.5%)	27.0 (4.5%)	
Age				0.551^1^
<25	78.0 (17.8%)	34.0 (20.9%)	112.0 (18.6%)	
26–40	49.0 (11.2%)	23.0 (14.1%)	72.0 (12.0%)	
41–60	181.0 (41.2%)	61.0 (37.4%)	242.0 (40.2%)	
>60	131.0 (29.8%)	45.0 (27.6%)	176.0 (29.2%)	
Employment status				0.648^1^
Full time	80.0 (18.2%)	24.0 (14.7%)	104.0 (17.3%)	
Part time	17.0 (3.9%)	9.0 (5.5%)	26.0 (4.3%)	
Retired	83.0 (18.9%)	32.0 (19.6%)	115.0 (19.1%)	
None	259.0 (59.0%)	98.0 (60.1%)	357.0 (59.3%)	
Coverage				0.493^1^
Cash	13.0 (3.0%)	3.0 (1.8%)	16.0 (2.7%)	
Good well fund	23.0 (5.2%)	10.0 (6.1%)	33.0 (5.5%)	
Ministry of Health	129.0 (29.4%)	57.0 (35.0%)	186.0 (30.9%)	
Prime Ministry of Jordan	64.0 (14.6%)	19.0 (11.7%)	83.0 (13.8%)	
Private insurance	25.0 (5.7%)	4.0 (2.5%)	29.0 (4.8%)	
Royal court	174.0 (39.6%)	65.0 (39.9%)	239.0 (39.7%)	
Cancer care program	11.0 (2.5%)	5.0 (3.1%)	16.0 (2.7%)	
Disease stage				0.320^1^
I	208.0 (47.4%)	75.0 (46.0%)	283.0 (47.0%)	
II	121.0 (27.6%)	39.0 (23.9%)	160.0 (26.6%)	
III	60.0 (13.7%)	32.0 (19.6%)	92.0 (15.3%)	
IV	50.0 (11.4%)	17.0 (10.4%)	67.0 (11.1%)	
Smoker				0.451^1^
Ex-smoker	73.0 (16.6%)	24.0 (14.7%)	97.0 (16.1%)	
No	292.0 (66.5%)	117.0 (71.8%)	409.0 (67.9%)	
Yes	74.0 (16.9%)	22.0 (13.5%)	96.0 (15.9%)	
Comorbidity				0.810^1^
No	270.0 (61.5%)	102.0 (62.6%)	372.0 (61.8%)	
Yes	169.0 (38.5%)	61.0 (37.4%)	230.0 (38.2%)	
Herbal medicine awareness and Knowledge level				<0.0011
Good	68.0 (15.5%)	71.0 (43.6%)	139.0 (23.1%)	
Moderate	202.0 (46.0%)	79.0 (48.5%)	281.0 (46.7%)	
Poor	169.0 (38.5%)	13.0 (8.0%)	182.0 (30.2%)	
Diagnosis year				0.605^1^
N-Miss	135.0	44.0	179.0	
Before 2010	11.0 (3.6%)	5.0 (4.2%)	16.0 (3.8%)	
2011–2015	18.0 (5.9%)	11.0 (9.2%)	29.0 (6.9%)	
2016–2019	55.0 (18.1%)	23.0 (19.3%)	78.0 (18.4%)	
2020–2023	220.0 (72.4%)	80.0 (67.2%)	300.0 (70.9%)	
Diagnosis				0.626^1^
Head and neck	29.0 (6.6%)	5.0 (3.1%)	34.0 (5.6%)	
Gastrointestinal	46.0 (10.5%)	19.0 (11.7%)	65.0 (10.8%)	
Urology	55.0 (12.5%)	18.0 (11.0%)	73.0 (12.1%)	
Orthopedics	19.0 (4.3%)	5.0 (3.1%)	24.0 (4.0%)	
breast cancer	133.0 (30.3%)	49.0 (30.1%)	182.0 (30.2%)	
Thoracic	12.0 (2.7%)	3.0 (1.8%)	15.0 (2.5%)	
Neurology	26.0 (5.9%)	14.0 (8.6%)	40.0 (6.6%)	
Hematology	65.0 (14.8%)	31.0 (19.0%)	96.0 (15.9%)	
Thyroid	20.0 (4.6%)	7.0 (4.3%)	27.0 (4.5%)	
Gynecology	17.0 (3.9%)	9.0 (5.5%)	26.0 (4.3%)	
Others	15.0 (3.4%)	3.0 (1.8%)	18.0 (3.0%)	
NA	2.0 (0.5%)	0.0 (0.0%)	2.0 (0.3%)	
Solid vs. Liquid				0.184^1^
Solid	375.0 (85.4%)	132.0 (81.0%)	507.0 (84.2%)	
Hematology	64.0 (14.6%)	31.0 (19.0%)	95.0 (15.8%)	

1: p-value was calculated by χ2 test.

The study compared the use of herbal medicine among 602 participants, where 439 (72.9%) were non-users of herbal medicine, and 163 (27.1%) were users, revealing significant differences in gender; females were more likely to use herbal medicine (68.7%), while males were less likely (31.3%). Married individuals were more prevalent among non-users (69.9%), while single individuals showed a higher proportion among herbal users (30.1%). Most participants reported earning less than 500 JOD, with secondary education being the most common level. The age group 41–60 years had the highest representation, with no significant difference between users and non-users (41.2%). The majority were not employed, with similar proportions between users and non-users (59.0%). The Ministry of Health (MoH) was the most common form of coverage for both groups. Most participants were in Stage I, with non-smokers being the majority. A majority had no comorbidities. A significantly higher proportion of users had good knowledge about herbal medicine compared to non-users (15.5%). Breast cancer was the most common diagnosis in both groups (Table 1).

### 3.1 Logistic regression analysis

The logistic regression analysis reveals that income levels, self-perceived herbal medicine awareness, and type of cancer (hematological vs. solid tumors) are significant predictors of herbal medicine use among cancer patients in Jordan. Patients with an income between 500 and 1000 JOD are about 3.29 times more likely to use herbal medicine compared to those earning more than 1000 JOD. Patients earning less than 500 JOD are about 3.18 times more likely to use herbal medicine compared to those earning more than 1000 JOD. Patients with “good” or “moderate” self-perceived herbal medicine knowledge are approximately 18.88 times more likely to use herbal medicine compared to those with “poor” knowledge. Patients with hematological cancers are about 2.24 times more likely to use herbal medicine compared to those with solid tumors. Other factors such as gender, social status, educational level, age, coverage, disease stage, smoking status, comorbidity, and employment status were not found to significantly influence herbal medicine use (Table 2).

**TABLE 2:** Predictors of Herbal uses among cancer patients in Jordan.

Predictor	Estimate	SE	Z	p	Odds ratio	95% confidence interval	
						Lower	Upper
Intercept	−4.1556	1.378	−3.0146	0.003	0.0157	0.00105	0.234
Gender							
M – F	−0.2198	0.31	−0.7092	0.478	0.8027	0.43724	1.474
Social status							
Married – Single	−1.007	0.533	−1.891	0.059	0.3653	0.12865	1.037
divorced – Single	−0.5486	0.924	−0.5936	0.553	0.5778	0.09441	3.536
widowed – Single	−0.2807	0.711	−0.3947	0.693	0.7553	0.18744	3.043
income levels							
1,000–500 JOD – More than 1000 JOD	1.1901	0.499	2.3832	0.017	3.2875	1.23534	8.749
Less than 500 JOD – More than 1000 JOD	1.1571	0.506	2.2865	0.022	3.1806	1.17968	8.575
Educational level							
Diploma – None	0.0242	0.851	0.0284	0.977	1.0245	0.19322	5.432
Higher education – None	0.7087	0.724	0.9783	0.328	2.0313	0.49113	8.401
Secondary or less – None	0.342	0.685	0.4994	0.618	1.4077	0.36783	5.387
Age							
26–40 – >60	0.5268	0.475	1.1089	0.267	1.6934	0.66745	4.297
41–60 – >60	0.0751	0.372	0.2016	0.84	1.0779	0.51958	2.236
< 25 – >60	−0.4857	0.687	−0.7068	0.48	0.6153	0.16001	2.366
Coverage							
Good well fund – Cash	0.6169	1.159	0.5322	0.595	1.8531	0.19114	17.966
Ministry of Health – Cash	0.7563	1.01	0.7486	0.454	2.1303	0.29411	15.431
Prime Ministry – Cash	−0.2505	1.067	−0.2347	0.814	0.7784	0.09608	6.306
Private insurance – Cash	−0.4031	1.169	−0.3448	0.73	0.6683	0.06761	6.606
Royal Court – Cash	0.399	1.013	0.3938	0.694	1.4903	0.20459	10.855
Cancer care program – Cash	1.2351	1.283	0.9625	0.336	3.4387	0.27803	42.531
Disease stage							
II – I	−0.6241	0.328	−1.902	0.057	0.5357	0.28159	1.019
III – I	−0.1607	0.371	−0.4329	0.665	0.8516	0.41144	1.763
IV – I	−0.3885	0.407	−0.9535	0.34	0.6781	0.30509	1.507
Smoker							
Ex – No	−0.0337	0.381	−0.0885	0.929	0.9668	0.45803	2.041
Yes – No	−0.0735	0.409	−0.1797	0.857	0.9292	0.41682	2.071
Comorbidity							
Yes – No	0.3148	0.308	1.0233	0.306	1.37	0.74964	2.504
Self-perceived Herbal medicine awareness and Knowledge level							
Good – Poor	2.9382	0.441	6.6689	<0.001	18.8809	7.96167	44.776
Moderate – Poor	1.9877	0.422	4.7056	<0.001	7.2988	3.18929	16.704
Employment status							
Yes – No	0.1906	0.356	0.5359	0.592	1.21	0.60258	2.43
Solid vs. Liq							
Hematology – Solid	0.8047	0.386	2.087	0.037	2.2361	1.05019	4.761

Note. Estimates represent the log odds of “Herbal usage = Yes” vs. “Herbal usage = No”.

### 3.2 Knowledge assessment among herbal users

The knowledge assessment among herbal users (N = 163) revealed diverse patterns in their use and perceptions of herbal medicine. The majority of patients reported daily use of herbal medicines (56.4%), with a significant portion believing that herbal medicines can help maintain health (54.6%) and even treat diseases (47.9%). However, a considerable percentage also expressed concerns about the safety of herbal medicines; 28.8% disagreed with the notion that herbal medicines are safe solely because they are natural. Interestingly, 50.3% of respondents disagreed that herbal medicines are safer than conventional medicines, indicating skepticism about their efficacy. A majority (58.9%) agreed that many of the health claims made by manufacturers and sellers of herbal medicines have not been proven, and 76.6% acknowledged a lack of available information on herbal medicine use (Table 3).

**TABLE 3:** Knowledge assessment among Herbal-users.

Knowledge area	*Overall (N = 163)*
Frequency of use	
Daily	92 (56.4%)
Monthly	30 (18.4%)
Weekly	41 (25.2%)
Herbal products can be used to help maintain and promote health	
Strongly Disagree	6 (3.7%)
Agree	89 (54.6%)
Strongly agree	42 (25.8%)
Disagree	26 (16.0%)
Herbal products can be used to treat the disease	
Strongly Disagree	13 (8.0%)
Agree	78 (47.9%)
Strongly agree	18 (11.0%)
Disagree	54 (33.1%)
I believe herbal products are safe because they are made from natural ingredients	
Strongly Disagree	9 (5.5%)
Agree	77 (47.2%)
Strongly agree	30 (18.4%)
Disagree	47 (28.8%)
If the herbal mix ingredients are available to the public, I am confident that it is safe	
Strongly Disagree	21 (12.9%)
Agree	46 (28.2%)
Strongly agree	14 (8.6%)
Disagree	82 (50.3%)
I think herbal products are better for me than conventional medicines	
Strongly Disagree	28 (17.2%)
Agree	35 (21.5%)
Strongly agree	15 (9.2%)
Disagree	85 (52.1%)
I believe that many of the health claims made by manufacturers and sellers of herbal products have not been proven	
Strongly Disagree	12 (7.4%)
Agree	96 (58.9%)
Strongly agree	27 (16.6%)
Disagree	28 (17.2%)
Do you agree that there is currently a lack of information available to you regarding the use of herbal medicines	
Strongly Disagree	5 (3.1%)
Agree	92 (56.4%)
Strongly agree	33 (20.2%)
Disagree	33 (20.2%)
Would you like to learn more about the safety and effectiveness of herbal medicines?	
Strongly Disagree	4 (2.5%)
Agree	83 (50.9%)
Strongly agree	55 (33.7%)
Disagree	21 (12.9%)
Do you know what happens because of interactions between herbal or alternative medicine with chemotherapy and medications?	
No	100 (61.35%)
Yes	63 (38.65%)
Were you informed about the effects of interactions between herbal or alternative medicine with chemotherapy and medications?	
No	105 (64.4%)
Yes	58 (35.6%)
Were you aware of the side effects of herbal or alternative medicine?	
No	81 (49.7%)
Yes	82 (50.3%)
At what stage of treatment did you start using herbal or alternative medicine?	
During treatment	27 (16.56%)
After treatment	21 (12.88%)
Before treatment	115 (70.55%)
Do you know the effect of herbal or alternative medicine on surgical outcomes?	
No	131 (80.4%)
Yes	32 (19.6%)
Were you informed about the impact of herbal or alternative medicine on surgical outcomes?	
No	139 (85.3%)
Yes	24 (14.7%)
Information Source of herbal	
Family	89 (54.2%)
Social media	88 (53.66%)
Friends	48 (29.27%)
Healthcare Providers	19 (11.5%)

Moreover, the data highlighted significant gaps in patient education, with 61.35% of respondents unaware of the potential interactions between herbal medicine and conventional cancer treatments, and 64.4% reporting that they had not been informed about these interactions by healthcare providers. Despite this, a strong desire for further education was evident, as 84.6% of participants expressed interest in learning more about the safety and effectiveness of herbal medicines (Table 3).

## 4 Discussion

The present study aimed to explore herbal medicine and its predictors of among cancer patients in Jordan, providing insights into the socio-demographic, clinical, and perceptual factors that influence this practice. Our findings align with and expand upon previous research conducted in various populations and settings, emphasizing the multifaceted nature of herbal medicine use among oncology patients.

Our study’s results are consistent with previous research in the Middle East that indicated a significant prevalence of herbal medicine usage among cancer patients. Results from a cross-sectional survey carried out in 2013 show that as much as 85% of the Arab population utilizes herbal medicines, a figure that significantly higher than 50% prevalence found in developed countries (Wazaify et al., 2013). In Arab nations, a wide variety of herbs have been utilized to treat various health conditions (Abu-Irmaileh and Afifi, 2003). Herbal medicine is one of the most frequently used alternative and complementary treatments among cancer patients in the region. For instance, in Saudi Arabia, 3% of cancer patients pursue complementary and alternative therapies, with herbal medicines being the main preference (Aldahash et al., 2012; Elolemy and Albedah, 2012). In Jordan, the rate of herbal medicines use is 35.5% among cancer patients (Abdel-Qader et al., 2020), whereas a greater percentage (60%) is noted in Palestine (Ali-Shtayeh et al., 2011). Additionally, breast cancer patients in Palestine show a greater prevalence of herbal medicine usage (68%) in comparison to those in Jordan (30.2%) as well as our study results (27%) (Jaradat et al., 2016).

Our findings highlight that income level emerged as a significant predictor, with patients in lower income are more likely to use herbal medicines. This association may reflect the perceived affordability and accessibility of herbal medicines compared to conventional treatments, especially in resource-limited settings. Similar patterns have been observed in studies conducted in other regions, suggesting that economic constraints often drive patients toward complementary and alternative medicine (CAM) options, including herbal treatments (Aboufaras et al., 2023).

Interestingly, the type of cancer (solid vs. hematologic) was also a significant predictor, with patients suffering from hematologic cancers being more inclined to use herbal medicine. This could be attributed to the chronic nature of these conditions and the prolonged treatment regimens, which might lead patients to seek additional therapies to alleviate symptoms or improve quality of life. Controversially, Asiimwe et al., in 2021 found patients with hematological malignancies (7%, 95% CI: 2%–16%) were less likely to report using herbal medicine in cancer treatment (Asiimwe et al., 2021).

Self-perceived knowledge and awareness of herbal medicine had a robust association with its use, indicating that patients who consider themselves well-informed are more likely to engage in herbal treatments. This highlights the importance of patient education in shaping health behaviors and the potential role of misinformation in driving the use of unproven therapies. The strong correlation between perceived knowledge and herbal usage underscores the need for healthcare providers to actively engage in discussions about CAM with their patients, ensuring that they have accurate information and can make informed decisions (Vasques et al., 2024).

Our findings align with global patterns of CAM usage, where socio-economic status, type of cancer, and patient perceptions are frequently cited as key determinants (Sadat Bazrafshani et al., 2019). However, the cultural context of Jordan, where traditional and herbal medicine holds significant historical and cultural value, adds a unique dimension to these predictors. This cultural backdrop may amplify the reliance on herbal treatments, especially among older adults and those with limited access to conventional healthcare services (Afifi et al., 2010).

The insights gained from this study have practical implications for oncology care in Jordan and similar contexts. Healthcare providers should be aware of the socio-economic and cultural factors that may influence their patients’ use of herbal medicines. By understanding these predictors, providers can better address the potential risks and benefits of herbal use, particularly in relation to interactions with conventional cancer treatments. Additionally, targeted education efforts could help mitigate the reliance on herbal medicines that may not be supported by scientific evidence, thereby improving patient outcomes (Aboufaras et al., 2023).

This study has several strengths. It fills a significant gap in existing literature. It uses a comprehensive approach, including socio-demographic, clinical, and perceptual predictors, to provide a holistic understanding of factors influencing herbal medicine use. The large sample size enhances generalizability to the broader Jordanian cancer patient population. The well-structured survey instrument offers nuanced insights into patients’ decision-making processes regarding herbal medicine use. However, the study is not without limitations. Its cross-sectional design limits causal relationships, and longitudinal studies are needed to establish causality. The reliance on self-reported data may introduce recall or social desirability bias, potentially affecting the findings.

Future research should explore the long-term outcomes of herbal medicine use in this population, as well as the potential interactions between herbal and conventional cancer therapies. By doing so, healthcare practitioners can better support patients in making informed decisions about their treatment options, ultimately improving overall care and patient outcomes in oncology settings.

## 5 Conclusion

This study provides valuable insights into the predictors influencing herbal medicine use among cancer patients in Jordan. The findings highlight that patients’ self-perceived awareness and knowledge of herbal medicine, income levels, and type of cancer significantly impact the likelihood of herbal medicine use. Interestingly, patients with higher self-assessed knowledge, lower income levels, and those diagnosed with hematological cancers are more likely to use herbal medicine. Additionally, the economic constraints faced by patients with lower income levels could drive them towards herbal medicine as a more accessible option. Understanding these dynamics is essential for healthcare providers to offer informed guidance to cancer patients, ensuring that their use of herbal medicine is safe, effective, and integrated with conventional treatment plans.

The main challenges in this context include addressing the lack of regulation surrounding herbal medicine use, ensuring that patients have access to accurate information, and overcoming cultural barriers that may hinder open discussions about alternative therapies. However, study results suggest that educational interventions targeting herbal medicine awareness may play a crucial role in shaping patients’ decisions to use alternative therapies, strengthen patient-provider communication, and create policies that promote safe and informed use of herbal medicines in oncology care.

## Data Availability

The raw data supporting the conclusions of this article will be made available by the authors, without undue reservation.
